# Remarks on Plethora, Read to the Medical Society of Tennessee, at Its Annual Meeting, in May, 1841

**Published:** 1841-07

**Authors:** Samuel Hogg

**Affiliations:** President of the Society


### WESTERN JOURNAL

OF

### MEDICINE AND SURGERY.

---

JULY, 1841.

---

ART. I.—*Remarks on Plethora, read to the Medical Society of Tennessee, at its annual meeting, in May,* 1841. By SAMUEL HOGG, M. D., President of the Society.

So often and so ably has the field of our profession been explored by the most eminent votaries of the science, that I have no hope of being able to bring forward any thing new or particularly interesting on the present occasion, and shall be happy if I can advance aught that has practical usefulness to recommend it. If, indeed, in the feeble state of my health, and under the trying bereavement which I have been called upon to endure, I had failed to address the Society at its present meeting, no one, perhaps, would have been disappointed. But anxious as I am to advance the interests of our profession, and to contribute all in my power to the usefulness of our meetings, I have resolved to offer a few remarks on a subject of much importance—I mean that state of the system denominated "Plethora,"—a condition, the prompt attention to which would prevent some of the most violent of acute diseases and subdue most of those of a chronic character. In submitting the following observations concerning it, I shall attempt to show the existence of plethora as a primary, tangible cause in the production or perpetuation of disease.

Plethora may be divided into two kinds: 1st., that which is natural, or as necessary to the developement, perfection and perpetuation of the whole organism of the system; and 2ndly., unnatural, or that which may arise from any deleterious cause acting upon the economy so as to derange its healthy operations. This may again be divided into general, or that which seems to affect simultaneously the whole system; and partial, involving one or but a few tissues, becoming a focus of irritation, and, if not removed, implicating, in time, every part of the body.

The first species, or natural plethora, prevails in the system of every healthy infant at birth. Few of the tissues, at that period, are perfect, but there seems to prevail, from the general laws which govern the economy, an exaltation of the vital properties commensurate with the important office they have to perform, in maintaining the growth of the system and perfecting the various organs. This state of plethora, when no disturbing circumstances interfere, is greatly under the influence of stated, septennary periods, developing and perfecting the different organs as they become necessary in the economy of the system. At seven months, for example, we see the first process of dentition showing itself in the child; at fourteen months the second; and at twenty-one months the third; at which time most children have the full number of milk teeth. We see, also, at times, during these periods, considerable aberrations from the regular order of things, as a want of balance in the circulation, irregular distribution of the nervous influence, and oppression of some important organ. Again, about the seventh year there seems to be a second revolution. The milk teeth are then removed, and a new set take their place; the mesenteric glands are more fully developed, and from that period to the fourteenth year, the development of the system is apt to progress healthily, unless great constitutional weakness exist, in which case these glands become diseased, and premature death is the consequence. In the south this is often the case. Children grow finely and promise well until about the sixth or seventh year, when they begin to decline and are carried off by tabes mesenterica, or some other visceral obstruction.

From the seventh to the fourteenth year, or the age of puberty, we witness nothing but the gradual growth of the child in stature and intellect; but about this period many highly important changes commence. Most of the great organs are perfected, and others are in progress towards maturity, making a demand upon the general capital exceeding that of any previous period. A rapid increase in growth commences, with increased susceptibility to the impressions of external agents, and an exhaustion of plethora in the tissues of supply, until about the approach of the next septennial period, when balance is established between them and the vessels of waste, and a state of the economy is the consequence similar to that in which man was originally formed. Although at the expiration of this latter period the organism may be perfect, and the system well balanced, yet from various causes, and particularly from the abuse of diet, drink, exercise, and sleep, and the excessive indulgence of the passions, the original excellence of the human organism has been lost, and a higher susceptibility at this period of our lives is awakened to various morbid influences.

After the third septennial period, so many additional causes are in operation, both moral and physical, that it becomes difficult to recognize the effects of the intervening periods until about the age of forty-two, when some failure generally begins to be visible in the animal functions. The individual becomes more intellectual; his ambition is now excited to the highest pitch, and among studious men, if the passion be indulged, is apt to weaken the organic functions and lead to impaired health. Hence we see so many of our young men of promise, at the present day, the victims of dyspepsia, and other chronic diseases which make such frightful waste in this class of our society. I will only add, on this branch of my subject, fearing lest I should tresspass on the patience of the Society, that the life of man has been limited to a septennary period of three score years and ten, instances of longevity reaching beyond that age being only exceptions to the general rule.

Hoping that the preceding remarks will be sufficient to engage your attention to what I have yet to propose, I now proceed to treat of the second branch of my subject, to-wit:

*Unnatural, or morbid plethora.* This species I have divided into general and partial. At the expiration of the third septennary period, as has been observed, there ought to be a perfect balance between the tissues of supply and those of waste; but we often find that, from some cause, the vital properties of the former remain so exalted as to maintain the want of balance, and produce a deposit of more than the necessary amount of fat to answer all useful purposes, constituting what is termed obesity, the most simple form of disease arising from this general plethora.

We often see persons who have been brought up in a plain manner acquiring a competency, and then quitting their laborious habits and taking to a sedentary life, become suddenly corpulent, with the appearance of high health. But if you congratulate them on the change, they will tell you that they do not feel well. They are troubled occasionally with fulness of the head, or wandering pains in the abdomen or joints, evidently arising from the full habit, the result of the change in the mode of life. Many such persons I have seen suddenly taken off by some structural lesion, without any premonitory symptom.

This state of the system I have found exceedingly common in the South. The facilities enjoyed by the man of industry and enterprise for growing rich in a short time are so great that he is often able to retire early in life from active business. He indulges freely in wine and the luxuries of the table; his excretions are not equal to the ingesta, and as a necessary consequence general fullness takes place, to relieve which he resorts freely to pills containing calomel as one of their ingredients. After a time these lose their effect, and beginning to feel more unwell, he applies to the doctor. He has now some pain in his shoulder or abdomen, with a soft tumor in the epigastric region just within the ilium, and slight soreness on hard pressure. What is it? His physician tells him it is "*liver disease;*" and as mercury is the specific, he must take calomel. He resorts to this Sampson, and as a large dose makes him feel better for the time being, the remedy must be repeated, whereby the liver is kept continually under the spur, until it becomes unable longer to resist its influence and structural lesions supervene, with dropsy of the belly as a consequence. I have often been consulted by persons laboring under this state of plethora, but have rarely been able, at first, to convince them that their disease was not one of the liver. From some experience in the treatment of such cases I am fully persuaded, that the liver is not diseased, and after a reasonable time I have generally succeeded in satisfying my patients of its soundness. What, then, is the matter, in such cases? I answer, the morbid condition is a general plethora, accompanied by complete engorgement of the cœcum, and I think the success of my plan of cure sustains this opinion. My first advice to such a patient is, to disgorge his pockets of the pills, and to quit his calomel—to disgorge his blood-vessels by the lancet, if any important organ seem to be threatened; and, finally, to disgorge his stomach and bowels, by strict abstinence, purgatives, or gentle laxatives, such as jalap or cream of tartar, calcined magnesia and the neutral salts, or magnesia and sulphur, by moderate exercise, and stopping his liquor. This course, if strictly adhered to, in a few weeks removes the plethora—the tumor and soreness in the abdomen give way, leaving a state of debility, which a prudent increase of diet, with regular exercise, in a few weeks more, entirely overcomes. If the liver should remain sluggish under this treatment, and the stomach continue to be disturbed under vitiated secretions, a mild alterative course of the blue pill, with alkalies, and some of the bitter astringents, will set all right and perfect the cure.

Every experienced practitioner must have observed the supervention of a most dangerous form of plethora about the favorable termination of chronic diseases, increasing the susceptibility of the great organs to such a degree, that the sudden application of any disturbing cause leads to sudden death. Many such cases have I witnessed, in which, after a hearty supper, the individual has been seized with sudden disease and died almost without a struggle.

Another form of disease arising out of repletion, attendant particularly upon chronic affections, and also frequently succeeding hemorrhages, seems to depend upon general plethora, and is curable at its onset, but ultimately is apt to terminate in some local lesion of structure if neglected. This occurs, also, after important surgical operations. This general plethora we also often find in old persons. They generally increase the amount of their diet with age, use more animal food, at the same time being unable to take as much exercise. Their great organs, as a consequence, become less active, and are constantly threatened with congestions under every violent external cause. They die more frequently under the same circumstances, than young persons, and chiefly for want of prompt depletion, and disgorgement of the viscera, the plethora, giving rise to local oppressions, being unfortunately mistaken for direct debility. I might cite many other consequences of this form of plethora, but deeming the foregoing sufficient to illustrate the subject, I proceed to consider the second species with its multiplied and dangerous consequences.

In considering the numerous diseases which come on as a consequence of this condition of the system, or which are aggravated by its existence, I know of no better plan than to take them in the order in which they occur in the different tissues, and as modified by the peculiar organization and vital properties of the tissues.

The first is the cellular tissue and the following are some of the affections which assail it:—phlegmon, carbuncle, furuncle, ulcers, anasarca, œdema, hemorrhage.

The next is the nervous tissue, which may be divided into that of animal, and that of organic life. It is of the first importance, and its diseases are more diversified and complicated than those of any other system. The diseases to which it is exposed are too well known to require a particular notice of them. Plethora is a frequent cause of them, and aggravates them whatever may be the cause which calls them into existence.

The vascular system, although simple in its functions, and subject to but few diseases, has nevertheless, a powerful influence in modifying the functions of the other systems. The veins and arteries are subject to inflammation, and the arteries to ossification, aneurism, contraction, and dilatation. The capillaries are most actively concerned in the various offices of the animal economy, and become involved in most of the diseases which assail the human body, plethora being the morbid condition by which they are perpetuated.

I might thus go on and speak of the exhalent and absorbent systems, of the osseous, the cartilaginous, the fibrous and the muscular systems, and of the mucous, serous, and glandular systems, with the disorders to which they are obnoxious; but I find that I must abridge these remarks, or I shall weary the attention of the Society. Plethora excited in any of these tissues becomes the cause of disease, and the great point in our treatment of them is to remove this morbid condition. I have said that, at birth, a natural plethora is a universal state, and this not being exhausted at the proper period, owing to accidental circumstances, the first care of the parent and of the physician ought to be to obviate the consequences of this failure. And this is to be done by a strict attention to the early physical and moral education of children, and by marriages so directed as to counteract any predisposition to disease that may exist. What better can we expect, as the product of the connexion of a man whose thoracic or abdominal viscera are predisposed to disease, or whose glandular system is not healthily developed, with a woman equally deficient in the same organs, than a feeble offspring requiring the care of both parents and physician, struggling on to puberty, there to dwindle and die, just as the fears of friends were beginning to be lulled? Instances of this sort, unfortunately, are familiar to all practising physicians, and they are of a nature so withering to domestic happiness that every man should be warned by them, in forming marriage connexions, to look for a companion in whom this want of balance, this predisposition to disease, does not exist. The principle extends to all the organs and functions of the economy, equally the instinctive and the intellectual. A defect in any organ is likely to be perpetuated in the offspring of the individual, and is to be countervailed by the selection of a partner in whom the defect is not found.

The primary physical and moral education of children necessarily devolving on the mother, as a preventive of the evils which result from plethora, she ought by all the means in her power to promote the healthy development of every animal and natural organ, throwing it upon its own resources as early as possible, that by proper exercise of its weaker parts they may be invigorated and the organs of the whole system be brought to a healthy balance. Strict attention to diet, both as to quantity and quality, I consider essential to the healthy development of the abdominal viscera. It should be the constant care of the mother never to suffer the stomach to be goaded by too great variety, or by such quantity of food as, by the frequent over-distension of that organ, may induce gluttony, and impair both its secreting and muscular functions, and thus lead to dyspepsia, with all its wretched train of evils. Except the abuse of ardent spirits, I know of few excesses so productive of mischief, or even so demoralizing in its effects, as excess in eating. Show me an epicure who indulges to excess in the luxuries of the table, and, as a general rule, you will find him a cold, calculating Shylock, destitute of every good feeling or sympathy, and bent only on his own profit or advancement.

By proper attention to diet and exercise during all the period of growth, I doubt not many of the deformities which we meet with might be prevented, and many operations for club-foot, strabismus, &c. be superceded. A vigor of constitution would also be imparted to the individual sufficient to sustain him under his intellectual labors, and prevent the many broken down constitutions which we daily see amongst the most promising men of our country. Permit me to relate two cases of congenital deformity which bear closely upon the subject under consideration: the first was that of a child with the patella in the ham. The nurse, in dressing it the second or third time after birth, discovered that it could not flex the left knee, which, on examination, was discovered to arise out of the cause alluded to. I was young in the practice, and the case was a novel one; but by bringing the dislocated bone around nearly to its place, and there confining it by a roller, I had the gratification of finding that nature overcame the defect, and that the child was put in possession of the perfect use of the limb. In the second case, a child eight or ten months old was subject to spontaneous dislocations of the shoulder. I ascribed the infirmity to a want of due antagonism between the flexor and extensor muscles of the humerus, and however false may have been my views of the pathology, the plan of treatment which I adopted in the case was entirely successful. The arm was drawn down to the side and kept in that position by means of a proper bandage, and the cold shower-bath applied to the shoulder, with frictions over the same part. The limb ultimately became strong and healthy under this treatment.

I will add, on the subject of the early education of children, that it equally concerns their happiness in life that a due balance be maintained between the various faculties of the mind. Much may be done by early culture to repress the strong and bring out the weak, and the character of the individual may thus be greatly modified and improved. With but this glance at a subject possessing the deepest philosophical and practical interest, I hasten on to the last part of my subject—the indications to be answered in the treatment of partial and general morbid plethora, and the diseases which owe their origin to one or the other of these states.

General morbid plethora being that fulness of habit which arises from the want of balance between the tissues of supply and those of waste, necessarily implies either an exaltation in the vltal properties of the former, or a failure of the latter to perform their functions. The first may be occasioned by excess of ingesta, causing oppression of the secreting organs and a failure in their functions. This form of general morbid plethora is the most simple, and points plainly to the proper treatment, viz: to lessen the ingesta both in quantity and quality; to avoid excitants; and, if danger of local congestions arise, to reduce the circulating mass either by general or local bleedings, and by occasional brisk purgatives. These, together with prudent exercise, aided by the recuperative powers of the system, will generally succeed in restoring the lost balance and in preventing the progress of the disorder to a more serious state.

The second form, or that arising from a failure in the function of the secreting organs, the tissues of supply not being inordinately excited, in most instances may be relieved by a mild alterative course of mercury, occasional gentle laxatives, and such specific remedies as excite the oppressed organ to more copious secretion. In the end, the preparations of iron may become necessary, with the bitter astringents, and in all cases, exercise, it must be remembered, is a remedy of the first importance.

Partial morbid plethora may arise from various causes, as sudden and violent impressions on the secreting tissues, or the frequent application of an agent which excites the system above the natural standard. If it be in a tissue not essential to life, the conservative powers of the system will remove it by resolution, effusion, or healthy suppuration. But if it be a tissue involving extensive sympathies, with functions important to life, a focus of irritation is set up, other tissues become involved, and general constitutional derangement is, at length, the result, demanding a course of treatment which will subvert this general irritative fever, promote the various secretions implicated, and remove congestion and its consequences. A judicious modification of the plan already proposed will generally accomplish this end.

I trust I shall not be deemed presumptuous in expressing my belief that here lies the great practical error of most of the French pathologists. They overlook that intermediate state of plethora, and the morbid effects of the retained elementary principles of the various secretions, which generally exist for a given time after the impress of the morbid cause, before phlogosis actually supervenes. Pursuing a negative, expectant course of treatment, a remora in the circulation takes place, with congestion and its consequences, acute or subacute inflammation; the susceptibility of organs is lost to the impress of such therapeutic agents as might have thrown off the offending excretions, disgorged the oppressed viscera, and, with the aid of the *vis medicatrix naturæ*, cured the patient. They do not appear to have drawn the distinction between the mild irritative fever resulting from this intermediate state of plethora, and that modification of morbid action, the result of phlogosis, which necessarily oppresses the vital powers to a degree which would forbid any further excitation. The Society will pardon this digression.

I will now proceed to the consideration of a few of the diseases arising out of local irritation of the different tissues which most frequently require the physician's attention; and hope to show that, in at least a majority of such cases, the treatment which succeeds will go far towards sustaining the opinions here advanced respecting the agency of plethora in producing and perpetuating them. In the cellular tissue we have phlegmon, ulcers, anasarca, &c.; in the nervous, convulsions, disorders of the senses, hysteria, hypochondriasis,

&c. The vascular system is obnoxious to inflammation and its consequences, contraction, ossification and its effects. In the capillary division of this system we note over-distension, suspended secretion, absorption, &c. The mucous, serous, and glandular systems are all subject to diseases arising from a depression of their vital properties, and a consequent failure in their ordinary functions, and retention of the elementary principles of their specific secretions. Now, suppose phlegmon to arise in the first named tissue, do we not, in its primary stage, attempt its cure by resolution, in other words, by a course of treatment that will lesson the vascular fulness, remove local irritation, and thereby prevent congestion and its consequences? Who at the present day would attempt the cure of dropsical effusions, except by the judicious use of the lancet, such medicines as promote active excretion, and, in the sequel, such tonics as are fitted to restore the organs to their normal condition, and aid the recuperative powers of the system in removing all disposition to plethora? From the nature of nervous diseases, and their effects on the animal economy, I am forced to believe that plethora of the investing membrane at their origin, or in the neurilema, must exercise considerable agency in producing and increasing them; and although powerful stimulants and antispasmodics occasionally act promptly as palliatives, still we find that, as a general rule, in this class of affections, the only radical cure is to be found in reducing the tone of the vascular system by prudent venesection, in allaying nervous irritation by appropriate remedies, in the judicious administration of such alteratives as excite the oppressed organs to free secretion, and in a regulated diet, and appropriate exercise.

I might go on to remark on the diseases of the other tissues, but I have said enough to illustrate the principle, and shall not trespass on your patience any farther. Let me observe, briefly, that it strikes me as a sound proposition in our profession, that the course of treatment which cures most generally and most promptly, under given circumstances, is the best; and that safety and success in practice are stronger arguments for the soundness of our theories than any which auscultation or post mortem examinations can supply. Not that I wish to disparage either of these valuable sources of information. The French pathologists have labored with a zeal worthy of all praise in these departments of medical science, and aided by the large institutions of Paris have thrown much light upon the changes in the human organism wrought by disease. But if they have not been rendered by the character of their researches too exclusive in their views, I am sure that the practitioner in this country, with limited opportunities for the use of the stethoscope and dissecting knife, is likely to be made so by depending too far upon the teachings of these instruments. He is apt to treat with contempt every fact and suggestion offered by those who have derived their knowledge at the bed-side, unless they happen to be in accordance with what he has been taught by his scalpel or stethoscope, and relies upon an expectant plan of treatment, confident that he will be able by post mortem dissections to verify his preconceived opinions.

In conclusion, I would remark that, in the treatment of disease, particularly in the Valley of the Mississippi, we should consider the system as a whole, duly appreciating the sympathies which subsist between all its parts, and avoiding the error of taking too limited or exclusive views in reference to the disorders by which it is assailed.

